# The Complexity of Procedural Fidelity in Precision Teaching: A Qualitative Analysis

**DOI:** 10.1007/s40617-025-01050-3

**Published:** 2025-04-02

**Authors:** Shauna Diffley, Richard M. Kubina, Chris Noone, Sinéad Quinlivan, Aoife Mc Tiernan

**Affiliations:** 1 School of Psychology, University of Galway, Galway, Ireland; 2 Department of Educational Psychology, Counselling, and Special Education, The Pennsylvania State University, University Park, PA USA

**Keywords:** Precision teaching, Procedural fidelity, Implementation errors, Qualitative methods, Frequency-building, Standard Celeration Chart

## Abstract

Precision Teaching (PT) is a system that involves precisely defining and measuring the frequency of behavior to facilitate timely and effective data-based decisions to accelerate behavioral repertoires (Evans et al., *Behavior Analysis in Practice,* *14*(3), 559–576 2021). When combined with instruction and practice approaches, PT has demonstrated efficacy in accelerating learning in academic skills. However, its use in public schools is minimal. One reason for this may be the limited amount of research exploring teacher-implemented PT and how PT fits within the context of busy classrooms. Procedural fidelity is an important concept to consider when procedures are implemented in complex and uncontrolled environments. The present study employed qualitative methods to explore expert practitioners’ and researchers’ perspectives on procedural fidelity, fidelity errors, and their perceived impact when implementing PT. The data were thematically analyzed and are discussed with regard to three overarching themes and their subthemes: (a) The Complexity of Procedural Fidelity in PT, (b) Maintaining Fidelity without Losing Flexibility, and (c) It’s a System. This study is an initial exploration of expert perspectives on procedural fidelity in PT, which can help inform future quantitative evaluations of the impact of fidelity errors on participant outcomes. Findings suggest that participants believed PT is a flexible system tolerant of some fidelity errors but that no component of the system should be omitted completely. Implications of these findings for research and practice are discussed alongside the strengths and limitations of this study.

Precision Teaching (PT), first coined by Ogden Lindsley in the 1960s, is a system that facilitates precisely defining and continuously measuring dimensional features of behavior and analyzing behavioral data on the family of Standard Celeration Charts (SCC) to make timely and effective data-based decisions to accelerate behavioral repertoires (Evans et al., 2021). Research has suggested that when used in conjunction with instructional and practice strategies such as frequency-building, PT is a highly effective system to accelerate learning and promote behavioral fluency across academic skills (Gist & Bulla, 2020; Mc Tiernan et al., 2021). That is, through its precise definitions of behavior, measurement, and display of frequency of behavior on a standardized chart, PT fosters responsive data review and decision-making that is associated with accelerated learner outcomes. The components of the PT system have been classified and defined by numerous researchers throughout its development (Johnson et al., 2021; Kubina & Yurich, 2012; Kubina et al., 2024) and recently updated by Evans et al. (2021) as (a) pinpoint, (b) arrange instruction or practice, (c) chart, (d) decide, and (e) try, try again. The system follows these components, but key to its conceptualization and application are its four underlying principles: (a) the learner knows best, (b) focus on observable behavior, (c) frequency as a measure of behavior, and (d) data displayed on SCC (Lindsley, 1990).

The first component, *pinpoint,* describes the approach to defining target behaviors for measurement within the PT system and offers an alternative to operational definitions. Using pinpoints in PT aligns with the system’s second underlying principle, namely, focus on observable behavior. Pinpoints follow the basic formula of an action verb (e.g., reads), an object or event that receives the action (e.g., word), and a context statement (e.g., from the book) (Kubina et al., 2024). They include a description of relevant learning channels (Haughton, 1980) and a pinpoint frequency aim. The learning channel specifies the sensory contact the learner makes with the stimulus/stimuli or input and the physical response modality of the behavior or output (Haughton, 1980). Following on with the above reading example, the learning channel would be ‘see word – say word.’ Frequency aims are performance standards denoted as range frequencies for a given skill that predict fluency outcomes, typically written as count per minute (Kubina & Yurich, 2012). A suggested frequency aim for the pinpoint see-say words on a page is 200–250 correct words per minute (Kubina & Yurich, 2012). This parallels a mastery criterion used in other behavior analytic approaches; however, a key difference is that mastery in PT is defined as the ability to perform behaviors accurately as well as at high frequencies (i.e., that behavior is performed fluently). The literature has numerous empirically validated frequency aims for a range of behaviors (Johnson & Street, 2013; Kubina & Yurich, 2012); however, frequency aims can also be determined through sampling performances of the behavior of skill experts or peers who are deemed as having mastered the behavior or skill.

When PT is used to improve academic skills in schools and education, it is implemented in conjunction with an instructional or practice approach to build fluency with the pinpointed behavior (or academic skill). The *arrange instruction or practice* component demonstrates that PT is a system, as it does not tell the intervention agent how or what to teach. The instructional approach should be selected by the intervention agent based on the evidence to best match the learners’ needs, preferences, and available resources, as well as permit the collection of frequency data (principle 3). Frequency-building is the most common approach used in conjunction with PT (Mc Tiernan et al., 2021) and refers to timed practice of a pinpointed behavior followed by performance feedback (Kubina & Yurich, 2012). Timed practice and performance feedback continue until the learner has achieved the selected frequency aim (or performance criterion).

Precision Teachers *chart* timed performances of pinpointed behaviors on the SCC. Frequency data obtained from timed practice is depicted on the standardized semi-logarithmic graph and can be interpreted in terms of performance (i.e., performance frequency or rate of behavior per minute) or learning (i.e., celeration or proportionate change in behavior over time). Both correct and incorrect performances are charted on SCC as independent data paths. Celeration values show how a given behavior has accelerated or decelerated across a span of weeks, months, or years (Kubina, 2019).

An advantage of charting celerations of “corrects” and “incorrects” is that a valuable “learning picture” develops. The *decide* component involves the examination of learning pictures to comprehensively analyze the data and make individualized decisions about the effectiveness of instructional approaches currently in place for the learner. Teachers, aides, parents, or the students themselves (i.e., chart “managers”) may decide to continue with the instructional approach or make a change. The decision to continue is typical when the learner is making timely progress toward their pinpoint frequency aim. This is demonstrated when the learner’s celeration slope demonstrates sufficient improvement in their performance over time. Alternatively, the decision to make a change occurs when the learner’s performance is (a) worsening, (b) stagnates and plateaus before they reach their frequency aim, or (c) when the learner has met their frequency aim. When a change in the instructional approach has not improved the learner’s performance, the precision teacher must *try, try again*. The fifth component emphasizes that PT is an iterative system guided by the principle “the learner knows best” and that the precision teacher will continually try new instructional approaches based on learning pictures until an improvement in performance has been achieved.

When learners achieve selected pinpoint frequency aims, they are said to have achieved fluency. Behavioral fluency is that combination of accuracy and a natural pace of responding that permits competent learners to function efficiently and effectively in their natural environments (Binder, 1996). Fluent behaviors are paceful, quick, automatic, flowing, flexible, effortless, errorless, masterful, and second nature to the individual learner (Johnson et al., 2021). The benefits of teaching to fluency include learners (a) *maintaining* skills after periods without practice, (b) *enduring* or performing the skill for longer than initially practiced, (c) performing the skill with *stability* even in distracting environments, (d) readily *applying* fluent skills in new environments, and (e) using fluent skills together with other fluent skills to *adduce* new blends of behavior (Johnson et al., 2021). It must be noted that there is some variation within the PT literature with regard to the terminology used to describe these benefits or fluency outcomes (Johnson & Street, 2012; Johnson et al., 2021; Kubina & Yurich, 2012).

There is evidence for the efficacy of the PT system in improving a range of academic skills, including reading, writing, math, and learning a second language (Gist & Bulla, 2020; Mc Tiernan et al., 2021). Along with the notable academic benefits, researchers report growth in students’ self-esteem, motivation, concentration, self-fulfillment, and work habits (Beck & Clement, 1991; Boyce & Najdowski, 2003; Griffin & Murtagh, 2015; Sundhu & Kittles, 2016). Despite evidence of effectiveness, the number of teachers and public schools using PT is minimal (Gist & Bulla, 2020). As highlighted by implementation science literature, establishing the effectiveness of an innovation does not guarantee its uptake into the natural environment (Bauer & Kirchner, 2020).

Mc Tiernan et al. (2021) summarized literature evaluating PT for improving academic skills. Of the 28 studies included in this systematic review, only seven employed teachers as intervention agents. Of those studies that employed teachers as intervention agents, an even smaller number provided information on teacher training. Four of the seven studies stated that teachers were trained, yet only one highlighted the content of training, and one other mentioned the intensity of training. Without additional information, critical features required for effective training in PT are unknown. Research investigating such critical features may be beneficial in increasing the uptake and impact of PT in public schools. In developing effective training for teachers as intervention agents, it is essential to acknowledge that adaptations are often necessary to increase the feasibility of interventions and to consider the relevance to context, such as population, class size, and resource availability (Owczarzak et al., 2016). However, with adaptations comes questions on procedural fidelity.

## Procedural Fidelity

Procedural fidelity refers to the degree to which independent variables are implemented as designed (St. Peter et al., 2023). This is an important concept when analyzing behavior, as it is necessary to reach accurate conclusions about functional relations between a procedure or intervention and the subsequent outcomes (Falakfarsa et al., 2022). Fidelity data, when collected in conjunction with learner progress, can help researchers and practitioners determine the extent to which errors impact learner progress. However, procedural fidelity is a complex topic to study with multiple associated variables that must be considered, including the type of fidelity measures (global vs. component fidelity), sequencing of fidelity levels, and type of fidelity errors (omission or commission errors).

To ascertain a thorough understanding of the impact of fidelity errors, one must ensure that they are exploring the type of fidelity most relevant to the given procedure. Global fidelity is the average fidelity across all components of a treatment package or procedure, whereas component fidelity is assessed on each component of the procedure (Cook et al., 2015). Component fidelity is critical in interventions with multiple components or in systems like PT, as each component may require differing levels of fidelity in order to serve as an active ingredient. Research has suggested increases in global fidelity may not represent increases in performance across all individual components, and performance on individual components can be highly variable (Abry et al., 2015; Cook et al., 2015). Researchers, trainers, and practitioners must understand a procedure or system’s active ingredients to create meaningful thresholds of fidelity (Abry et al., 2015).

Learner outcomes are typically best when an intervention is implemented with perfect fidelity. Although 100% fidelity is ideal, it is not necessarily achievable in nonexperimental settings like busy classrooms. In a systematic review of the literature, Brand et al. (2019) highlighted that 100% fidelity may not always be necessary to see positive intervention outcomes, with some interventions maintaining efficacy when fidelity was reduced to as low as 50% (St Peter Pipkin et al., 2010; Vollmer et al., 1999) and others when reduced to 25% (Northup et al., 1997). However, research has yet to determine the specific level of fidelity of individual components that need to be achieved before intervention outcomes are adversely affected (Brand et al., 2019), and this may vary depending on the intervention (McIntyre et al., 2007) and its context.

Parametric analyses of fidelity are the most adopted approach to explore the impact of procedural fidelity on intervention outcomes. They involve experimentally varying fidelity in order to analyze the effects on intervention outcomes. Parametric manipulations of fidelity are designed to resemble common fidelity errors that occur in practice. They can include implementing components that are not prescribed in the intervention protocol, referred to as commission errors, or omitting components that are prescribed, referred to as omission errors. Research on the types of fidelity errors is emerging, and future research is warranted on the effects of the different error types (Carroll et al., 2013).

Parametric manipulations of fidelity typically vary fidelity on one specific intervention or intervention component at a time, including differential reinforcement of alternative behavior (Northup et al., 1997; St Peter Pipkin et al., 2010; Vollmer et al., 1999), timeout (Northup et al., 1997), prompting procedures (Wilder et al., 2006), and reinforcement (DiGennaro Reed et al., 2011). However, parametric manipulations of fidelity are more challenging when behavior analytic procedures have multiple components. As demonstrated by Carroll et al. (2013) before conducting parametric analysis of fidelity in multicomponent procedures like Discrete Trial Instruction (DTI), steps must be taken to develop an understanding of procedural fidelity as it relates to that system or procedure in practice. Carroll et al. conducted a descriptive assessment to develop an insight into fidelity errors in DTI and to identify components on which to vary fidelity. An alternative approach to descriptive assessments to provide insight into fidelity may be found in qualitative research.

## Qualitative Research

Qualitative analyses provide one avenue to explore complex topics such as procedural fidelity in systems like PT. Although a highly valued methodology in other fields, literature, including qualitative analysis in behavior analytic and PT research, is scarce. However, recent research has argued the case for qualitative analysis in behavioral research, stating that qualitative investigations may allow behavioral researchers to broaden the type of research questions they ask (Burney et al., 2023). This is exemplified by the work of St. Peter et al. (2023), who explored behavioral scholars’ perspectives on reporting procedural fidelity.

Researchers in the fields of education and implementation science have recognized the importance of qualitative methods in bridging the research-to-practice gap and provided a rationale for the methodology used in the current study. Qualitative research can play an important role in shaping education policy and practice by illustrating the messy realities of schools and facilitating important discourse that prompts school practitioners to change and adjust their practices (Kozleski, 2017). Qualitative methods also provide information on how and why efforts to implement best practice may succeed or fail and how patients and providers (learners and intervention agents in the case of PT) experience and make decisions in practice (Hamilton & Finley, 2019). For example, Cohen et al. (2008) used qualitative methods to explore how interventions change during implementation in ten interventions designed to improve health promotion in primary care practices. Similarly, Owczarzak et al. (2016) used qualitative interviews to explore how staff interpret and enact implementation fidelity with the need for adaptation in real-world delivery of an HIV prevention program. Thus, qualitative research offers an approach to exploring the concept of procedural fidelity as it relates to PT implemented in practice and may be used to inform future parametric analysis of fidelity in PT.

## Current Study

The current study employed qualitative methods to explore procedural fidelity within PT informed by implementation science literature as discussed. Specifically, the current study aimed to explore expert practitioners’ and researchers’ perspectives on (a) the meaning of fidelity errors in the field of PT, (b) common fidelity errors witnessed when training and supervising teachers to use PT, and (c) perceived impact of common fidelity errors on outcomes for learners.

## Method

### Participants and Recruitment Procedures

A purposive sampling strategy was employed. Eligible participants were required to meet the following criteria: (a) be practitioners or researchers in the field of PT, (b) have previously trained and supervised others to use PT in schools, (c) be older than 18 years old, and (d) be proficient in speaking English. Participants were recruited via email by the primary researcher. An initial list of potential participants was developed in line with the inclusion criteria via social media pages with a focus on dissemination of PT, centers/services known to implement PT in practice, academics who have published in the area of PT, and experts known to the research team. As the PT community is small, the email addresses of all possible participants were known to the research team. The initial email contained a participant information sheet and consent form. Individuals interested in participating were requested to return the consent form to the primary researcher via email.

An approximate dataset size of between four and 10 interviews was determined a priori based on information power (Malterud et al., 2016) and previous literature exploring the perspectives of trainers and supervisors in other fields. However, no exact dataset size could be determined owing to the qualitative nature of the project. Previous researchers exploring the perspectives of trainers and supervisors in radiography, adoptive parenting, and counseling have conducted interviews with four (Zorn et al., 2019), six (Bergsund et al., 2018), and ten (Gazzola et al., 2014) trainers/supervisors, respectively. On the basis of recent recommendations against saturation and in favor of information power as a determinant of dataset size (Braun & Clarke, 2021b), Malterud et al. (2016) information power estimation was used. Malterud et al. (2016)’s estimation is based on the following variables: (a) study aim, (b) sample specificity, (c) use of established theory, (d) quality of dialogue, and (e) analysis strategy.

We proposed a small dataset size was required as this study has (a) a narrow study aim, (b) a highly specific sample from the small PT community, (c) there is some established theory in the broader field of behavior analysis regarding the impact of fidelity errors on learner outcomes, (d) the researcher was expected to have a high quality of dialogue due to personal experience using PT and familiarity with members of the community, and (e) the researchers aimed to use a cross-case analysis. A final dataset size of nine was achieved. The first author, who also served as the interviewer, stopped recruiting when they believed that they had enough rich data to answer the research questions the study aimed to address.

Participants ranged in age from 27 to 71 (*M* = 42.67, *SD* = 11.77). Five participants identified as female, and four identified as male. Participants resided in England, Iceland, Italy, Northern Ireland, the United States of America, and Wales. Five participants used PT in both research and practice, three used PT in practice only, and one used PT in applied research only. Participants had an average of 14.44 years of experience with PT (*SD* = 12.23), ranging from four to 44 years. Participants had experience in training individuals from a range of different backgrounds to use PT, including teachers (*n* = 7), behavior analysts (*n* = 6), psychologists (*n* = 3), parents (*n* = 3), speech and language therapists (*n* = 2), teaching assistants (*n* = 2), sports coaches (*n* = 1), occupational therapists (*n* = 1), and animal trainers (*n* = 1). Participants had experience training individuals to use PT with school-aged children, some of whom were neurotypical, autistic, or had an intellectual disability. Participants typically conducted training through university programs (*n* = 5), in schools (*n* = 3), at conferences (*n* = 3), through their company training (*n* = 2), in homes (*n* = 1), and online (*n* = 1).

### Data Collection

Nine individual semi-structured interviews were conducted virtually over Zoom by the first author over a three-month span. Interviews lasted between 30 and 79 min and followed an interview guide. Informed consent and permission to record were obtained from all participants prior to interviews. The transcription feature on Zoom was used, and the transcripts created were used as a foundation for final transcripts. The first author listened to the audio and edited the initial transcripts until verbatim transcripts were complete. Recordings were deleted upon transcription, and transcripts were anonymized by removing any identifying information. NVivo software was used to assist in organizing the data into codes, themes, and record memos. Prior to the interview, demographic information was collected through Qualtrics, an online survey platform. The project was approved by the university’s research ethics committee and preregistered on the Open Science Framework. Anonymized transcripts, including interviewer questions and interviewee responses, can be found on Open Science Framework at (https://osf.io/n9q8z/).

#### Interview Guide Development

An interview guide containing open-ended questions (Appendix A) was developed by the authors based on the scientific literature on PT and procedural fidelity. Participants were asked about their experience of PT before exploring procedural fidelity and fidelity errors in practice in more depth. Participants were questioned about error topography, error prevalence, and error impact as it relates to their practice. Participants were asked about errors in general but also specifically related to the components of the PT system influenced by the component and global fidelity literature. Questions about eliminating components or adding other elements were asked to gather information on omission and commission errors.

### Data Analysis

This was a cross-case qualitative study in which semi-structured interviews were used to explore expert practitioners’ and researchers’ perspectives on procedural fidelity and common fidelity errors that occur when using PT. Template analysis, as per King and Brooks (2016), a “codebook” approach to thematic analysis, was used to analyze the data. This method was selected owing to its position within the qualitative paradigm as “qualitative pragmatism” (Braun & Clarke, 2021a). The research was driven by pragmatic demands of predetermined information required on fidelity errors in PT, along with the need for teamwork and reliability checks. The template approach to thematic analysis aligns with the first author’s pragmatic epistemology and critical realist ontology as coined by Heeks et al. (2019) “pragmatist-critical realism.” The first author used the abductive approach favored by pragmatist-critical realism when coding the data, which involves using both inductive and deductive coding in their analysis, moving back and forth between theory and data (Heeks et al., 2019). Deductive codes were developed on the basis of scientific literature and employed in the initial a priori codebook. The a priori deductive codes included (a) omission error, (b) commission error, (c) pinpoint error, (d) setting aim error, (e) recording error, (f) decision-making error, (g) try, try again error, (h) performance feedback error, and (i) impact on learner outcomes. Inductive coding was employed as the researcher analyzed the raw data. Data was analyzed in an iterative process in accordance with the phases recommended by King and Brooks (2016, 2018) and outlined in Table 1 below.

**Table 1:** Phases of template thematic analysis

Phase name	Description of phase application in current study
1. Generate initial codebook	An initial codebook was developed by the authors prior to data collection. The codebook was developed based on the research questions and the literature on PT and procedural fidelity as discussed in the introduction
2. Familiarization with data	Once data was transcribed, the first author and fourth author read through the data and familiarized themselves with the content
3. Preliminary Coding	The first author engaged in preliminary coding of five interview transcripts. A diverse sample of interviews were selected for analysis. The a-priori codebook was applied simultaneously with open coding in which new codes were created for any unexpected information that had the potential to aid the interpretation of the data and related to study aims, objectives and questions. The researcher coded inclusively and codes that were not relevant were discarded or merged with other codes later
4. Clustering	A-priori codes that had relevance to the data and newly generated codes were grouped together into initial clusters, redundant codes were removed, and codes were defined more precisely. Clustering began the process of initial theme development
5. Developing initial template	Once confident with initial clusters the first author explored the hierarchical relationships between codes and recorded these within the initial template, with child codes slotting within parent codes
6. Modify and develop the template	Two independent coders (The first and fourth authors) then applied the coding template 1 to a portion of dataset to assess its fit to the data. The first author applied the template to all nine interviews and the fourth author applied it to two. While the two independent coders applied template 1, they annotated data that did not fit within the current template. Template 2 was developed based on both coders discussion of the annotations they made while applying template 1 and based on the coding comparison query. This included creating new codes, redefining codes or dropping codes, creating hierarchical relationships and restructuring some initial relationships. However, the new template was not applied to the full dataset each time a modification was made. Template 2 was applied to another two interviews by both coders. Template 2 revealed no new codes, and the template provided rich representation of their data. Coding definitions were clarified and agreed by both coders, and this represented the final template
7. Final Template	The final template was applied to the full dataset by the first author only
8. Interpretation	The first author then used the data coded in the final template to interpret meaning in relation to the research questions and begin generating themes. Theme development was a recursive process. The first author explored the initial themes and hierarchical relationships generated during the clustering phase. The first author engaged the technique of visually mapping or generating a series of thematic maps to help them with theme development and explore the pattern between themes, subthemes and codes. When the first author developed potential themes, they reread the dataset to see if the themes represented the key concepts the data portrayed, reviewed research questions and brought themes to the fifth author for review. Both authors repeated this process reviewing, redeveloping and refining themes until themes portrayed a true representation of the dataset and addressed the research questions
9. Report	The authors wrote a report explaining their interpretation of the data in relation to the study aims and research questions (see results)

#### Researcher Reflection

The data was thematically analyzed by the first author using template analysis. The fourth author analyzed four transcripts for the purposes of inter-coder reliability. The fifth author contributed to the interpretation of the template thematic analysis. As researcher subjectivity is key to good qualitative analysis (Braun & Clarke, 2021b), the authors involved in the analysis wish to acknowledge how their learning history has shaped the analytic process.

The first author is a white Irish female with four years of experience in behavior analytic research. The author could be considered an “insider researcher” in relation to these data. An insider researcher as per Braun and Clarke (2021b, p. 18) is “in some or many ways a member of the group they are studying (e.g. sharing a racial or ethnic heritage or particular life experience with the participant group).” PT is the first author’s primary area of research. As an insider researcher the interview topic is familiar to the first author. She recognizes the importance of reflecting on this to ensure that familiarity with the topic does not shape the analysis entirely. Working with a research team, all of whom bring different personal and academic learning histories to the data, provides the first author with an opportunity to check in and reflect on the impact of their learning history in shaping the analysis.

The fourth author is a white Irish female residing and practicing in Australia with four years of experience in behavior analytic research and practice. The author may be considered an outsider researcher in relation to these data as she has a basic understanding of PT but a rudimentary knowledge of the topic in general to date. Currently, the fourth author holds a role as a Board-Certified Behavior Analyst (BCBA) supervisor in a center that provides behavioral intervention and support to children, where she has experience in training those without a background in behavior analysis, including teachers in behavior analytic technologies and using fidelity data within training contexts.

The fifth author is a lecturer in Psychology, specifically in Behavior Analysis, with extensive experience in conducting research in the area of PT, as well as working alongside and training teachers in behavior analytic interventions and supports. Therefore, the fifth author could be considered an insider researcher in that they have a direct connection to the research questions related to their experiences in training others to implement PT. However, the authors’ experience in training teachers in school settings to implement PT has predominantly been within a research context with an aim of collecting objective data and controlling for bias.

#### Intercoder Reliability

To ensure the fit of each template to the data and foster reflexivity, intercoder reliability testing was conducted. Although intercoder reliability is not recommended in some types of qualitative analysis, such as reflexive thematic analysis (Braun & Clarke, 2021a), approaches such as template thematic analysis do lend themselves to intercoder reliability. It is important, however, to note that such statistics should be interpreted with caution, as quantitative statistics are not fully representative of coding reliability in qualitative research methods. For example, automated intercoder reliability tools are “overly sensitive to inconsequential differences in coders” files (e.g., when coders have selected data units that differ by a mere punctuation mark) (O’Connor & Joffe, 2020b, p. 5). Intercoder reliability statistics were merely used to guide coding decisions and foster dialogue and reflexivity among the coders (O’Connor & Joffe, 2020a).

The first and fourth authors applied each template to a subset of the data. Typically, intercoder reliability is conducted on 10–25% of data (O’Connor & Joffe, 2020a). For the purpose of the current study, two transcripts (22%) were coded by the first and fourth author for each revision of the template, i.e., four in total. A coding comparison query was conducted on NVivo, and percentage agreement and Cohen’s kappa were reviewed. For template 1, percentage agreement was 97.85%, and Kappa was 0.41, indicating fair to good agreement. On application of template 2, the percentage agreement was 97.20%, and the Kappa was 0.54, which indicated a fair to good agreement. After each application, the first and fourth authors met and compared and discussed the analysis. In the case of opposing explanations, the first and fourth authors discussed until 100% agreement was reached. Similarly, when template modifications were necessary, consensus building among team members ensured that both authors were in agreement on the current template.

## Results

**Fig. 1:**
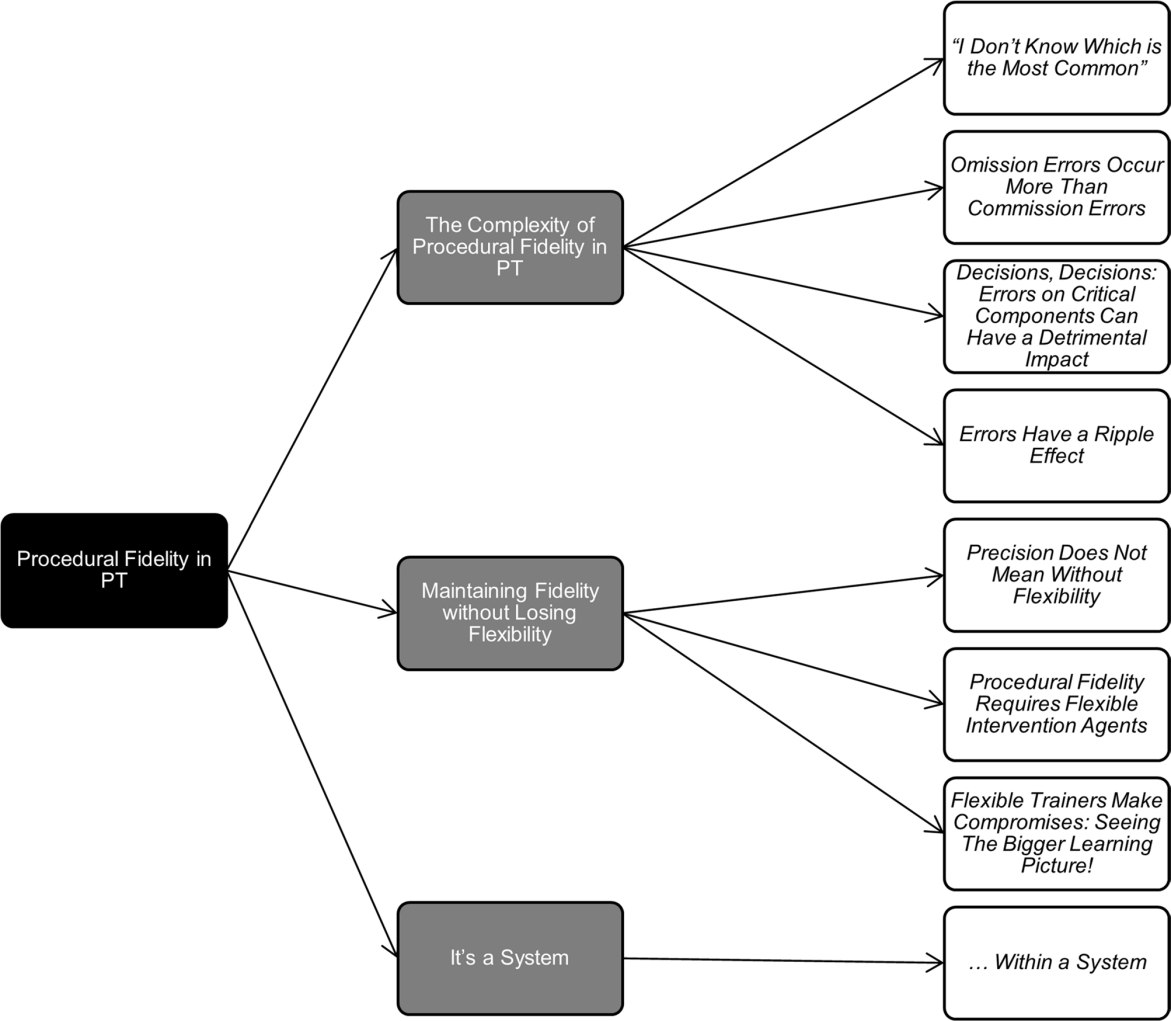
Thematic map

The interviews provided a rich and meaningful insight into the participants’ understanding and experiences of procedural fidelity as it relates to the implementation of PT in school settings. The authors used template thematic analysis to assist them in addressing the study aims. However, the authors must acknowledge that this thematic analysis goes beyond the initial study aims to include valuable information gathered regarding how to achieve high procedural fidelity and shield against fidelity errors in PT. Participants’ views on the matter have been conceptualized into three overarching themes summarized in Table 2 and depicted in relation to their subthemes in the thematic map in Fig. 1 below.

**Table 2:** Summary of themes

Theme Name	Description
The Complexity of Procedural Fidelity in PT	Begins to portray participants understanding of the meaning of procedural fidelity and fidelity errors in PT. It highlights the intricate nature of procedural fidelity in PT and the various errors that can arise across different components of the system
Maintaining Fidelity without Losing Flexibility	Encompasses interviewee’s thoughts on adaptations and variations when teachers use PT in practice and explores the parallel between lower fidelity and flexible components
It’s a System	Captures participants’ views toward PT being more than an intervention and that it is a complex and iterative approach to learning that must fit within co-existing systems and structures such as within a school

### The Complexity of Procedural Fidelity in PT

This theme portrays participants’ views on the meaning of procedural fidelity and fidelity errors in PT. It highlights the complexity of procedural fidelity in PT and the numerous different errors that could occur within each component of the PT system. The theme also displays the idiosyncrasies with regard to participants’ perspectives on fidelity errors and their impact.

#### “I Don’t Know Which is the Most Common”

Participants described a wide range of fidelity errors observed while training others to implement PT and the difficulty in determining the single most common error. *Pinpoint*, *chart*, and *decide* were the components that were emphasized when exploring errors observed in practice across interviews. Participants highlighted that teachers often struggled to *pinpoint* the behavior they wanted to measure or to select the most appropriate skill to pinpoint. With regard to the *chart* component, participants talked about intervention agents regularly having difficulty with more advanced elements of charting, such as *“charting the record floor? […] not charting the data by minute.*” Record floors are symbols used on the SCC to denote the duration of a timing period and represent the lowest non-zero result of an assessment (White & Neely, 2012). Another common error included intervention agents *“charting after the fact,”* meaning that they chart data post-session, which in turn prevents in-session decision-making. Failing to change the instructional or practice approach when learners are not progressing was most commonly observed within the *decide* component.

There were some differing views among participants with regard to the frequency of certain errors on the *chart* component. Some participants believed incorrectly charting data, i.e., plotting corrects and incorrects on the SCC, was one of the most common errors, while most participants believed that it was one of the least common and stated that when intervention agents do chart, they chart quite well.

*“I think people, whoever all the trainers out there are, they get the people to accurately plot the data, in my experience. Unfortunately, that's too much of a focus sometimes.”*

Similarly, participants spoke about a range of errors that occur on a less frequent basis. Some participants stated that forgetting to measure incorrect responses or learning opportunities occurred but was less common. Another mentioned that they had occasionally observed timing periods that did not match the pinpoint, e.g., timing for too long.

#### Omission Errors Occur More Than Commission Errors

When interviews were analyzed for errors of omission and commission, participants most frequently mentioned omission errors. Most omission errors highlighted were in relation to the *chart* and *decide* components. Numerous participants highlighted that the SCC is often omitted completely and that more *“advanced elements”* of the chart are often omitted, including record floors and minimum celeration lines. Minimum celeration lines are used as a means of setting goals and are drawn on the chart to indicate the minimum rate of progression that the learner needs to make to meet their pinpoint frequency aim in a timely manner. Similarly, participants mentioned that the *decide* component is often completely omitted.

*“Not making changes according to the data. So even the data is kind of showing that you're kind of getting either a very flat line or minimal celeration, just kind of sticking with what you're doing and not changing anything up whatsoever.”*

Although less frequent, commission errors were also discussed. They included adding “*non-behavioral verbs*” to pinpoints such as *“know, understand, or be able to,”* as well as calculating the percentage of correct responses and the use of verbal feedback or coaching during timed practice. Making decisions too often was a commission error mentioned by several participants. One participant described it as “*they [intervention agents] make so many decisions rapidly we can't assess the effects of an intervention because it’s like change, change, change, change*.”

#### Decisions, Decisions: Errors on Critical Components Can Have a Detrimental Impact

When talking about fidelity errors, participants without guidance regularly referred to the impact that such an error would have on learners and particularly emphasized emotional impact.

*“It just hurts my heart seeing a child or a learner of any age on the same skill for weeks, and they’re not doing any better. Like, talk about deflating motivation.”*

Most participants believed errors in the *decide* component were the most detrimental. Specifically, failing to change the instructional or practice approach when learners are not progressing was highlighted as the most damaging error in this component.

*“The children themselves get to a point where they’re like, oh, do you know what? I just can’t do this. I’m not gonna do it anymore. I’m not getting any better, you know. So and so is on this pack* (level)*, and I’m not getting anywhere, or I’m really bored of this now.”*

Participants emphasized that failing to change the instructional or practice approach when learners are not progressing also impacts the intervention agents and trainers. One participant remarked, *“Whoever is implementing the program also may […] kind of, you know, not believe in it as much.”* Participants also considered the absence of a session routine and starting practice with skills before accurate responding was achieved as detrimental to the effectiveness of the system.

Participants reported that they believed the least detrimental errors were those associated with specific or more advanced charting conventions. One participant emphasized this by saying, “*I don’t really know when charting zero if it makes a difference if you use a question mark, divide by two below the counting time, or an upside-down question mark*.” Another said, “*If I would be confident that the employee would be like consistent in always charting the data by minute, but then I wouldn’t see like a record floor. I would not be super concerned.*” One participant believed omitting the chart completely was not overly damaging, whereas most stated that the chart was necessary, but some variation and errors in charting conventions could be tolerated. Participants also reported that variation in the amount of coaching used during frequency-building timed practice was one of the least damaging errors they have seen in practice.

#### Errors Have a Ripple Effect

Multiple participants perceived *pinpoint* errors as having a damaging impact and a knock-on effect on all other components. One participant described this as “*if you pinpoint the wrong thing, you can’t be sure […] if the behavior change you’re getting is what you need.*” *Pinpoint* was not the only component on which errors were perceived to cause a ripple effect. Participants reported that errors on the *chart* component led to errors on the other components of PT, as it affects one’s ability to analyze the data. Errors on the SCC could lead the intervention agent to believe that a learner’s performance is better than it is and to continue with an instructional approach when a change is warranted or vice versa. Participants highlighted that errors with regard to setting pinpoint frequency aims can have a ripple effect, impacting the sustainability of the PT system. Setting frequency aims too high may prevent both teachers and students from contacting reinforcement. One participant said, *“They won’t really see the effects that they want, […] and they’ll go, do you know what, I’ve been doing this for 3 weeks, it’s not working, I give up”*. Without a strong reinforcement contingency early on, both teachers and learners may become frustrated, which may lead to more fidelity errors or even complete disengagement with PT.

### Maintaining Fidelity Without Losing Flexibility

Participants acknowledged that the components and principles of the PT system were fundamental but believed that some flexibility in their application could be tolerated. In some cases, flexibility was emphasized as essential for the successful implementation of PT. Flexibility was conceptualized as the adaptability of a component, intervention agent, or trainer in PT. Flexibility is discussed as a spectrum from flexible to inflexible.

#### Precision Does Not Mean Without Flexibility

Participants reported that some components of the PT system are flexible and more tolerant of fidelity errors than others. Participants discussed the flexibility of all components of the PT system, apart from *try, try again*. The majority of participants discussed elements of the *chart* component within which flexibility in implementation could be tolerated. The regularity with which one charts data was highlighted as a flexible element. How often one charts can vary since there is a family of charts from which to choose. Although the daily per minute SCC is the most used, intervention agents have the option to use weekly, monthly, or yearly SCC.

“*Chart weekly and then make decisions on a weekly basis. At least you’re still regularly making decisions.*”

Some charting conventions were also highlighted as being flexible, with one participant summarizing the level of flexibility they believed should be tolerated as “*the conventions can be a little looser […] As long as everybody is clear about what everything means, I’m cool.*”

Several participants believed that there is flexibility regarding how pinpoint frequency aims are set. Some participants reported that it did not matter whether frequency aims were set based on research or determined by measuring a fluent performer’s rate of responding. Two participants highlighted *arrange instruction or practice* as a flexible component, specifically, for how long one engages in practice and how often practice sessions are conducted. One interviewee highlighted this by saying, “*We came to a compromise, that they do it 3 days a week […], but one of those sessions should ideally be on a Friday […], and that seems to work quite well.*” Another participant highlighted aspects of the *decide* component that offered flexibility. They noted that if intervention agents make.

“*If your change in an intervention is ineffective, that’s not really a problem, right? You just change it again. It tells you pretty quick, so that probably wouldn’t greatly impact if you’re looking at your data frequently enough*.”

Others discussed the essentiality of flexibility within some components. Within *arrange instruction or practice*, flexibility was discussed when implementing frequency-building. In particular, one participant highlighted the flexibility necessary to implement performance feedback since it should be individualized for each learner and dependent on the intervention agent. In other words, every intervention agent has a slightly different approach to providing feedback in line with their personality.

Although participants emphasized the flexibility of the system, they also reported that some components are inflexible and require higher levels of fidelity. Participants believed that the *decide* component, specifically continuous data review, was the most inflexible component and must be implemented to the highest level of fidelity. Participants believe that intervention agents should review the learning picture as they chart.

#### Procedural Fidelity Requires Flexible Intervention Agents

While conveying the message that being a flexible intervention agent is important, participants discussed how inflexibility can often present itself in practice. Participants described inflexible intervention agents as procedurally rigid, and as a result, the PT system gets “*all kind of rote feeling.*” Participants hypothesized one reason intervention agents become inflexible is that following a set procedure is simply easier.

“*The biggest implementation problem is getting folks to move away from a procedure […] People like black and white procedures.*”

Another reason for the inflexibility in implementing PT may be a lack of conceptual understanding. This presents as intervention agents lacking versatility when making data-based decisions and making changes to instructional and practice approaches.

*“Just very rigidly sticking to… basically not making decisions based on the data, just sticking to very rigid kind of fluency building.”*

Participants believed that a strong conceptual understanding may prevent fidelity errors and promote flexibility in intervention agents. Participants identified key concepts, including rate, fluency, agility, *“growth is nonlinear,”* and sequencing as essential for intervention agents to understand. Such conceptual knowledge may develop their flexibility. As one participant stated, *“If they could really learn those concepts, I think the whole thing would make a lot more sense to people. And when things make more sense to people, they don’t need like 85 steps.”*

Participants also discussed how a lack of conceptual understanding can lead to more fidelity errors and rigidity, such as PT being used where it is not suitable, including efforts to increase fluency before a learner demonstrates accuracy. Additionally, a lack of conceptual understanding and flexibility can result in a negative implementation experience for the intervention agent.

*“It gets, it’s very tedious. It feels tedious as a trainer, and it feels tedious as a learner. I think that doesn’t work.”*

Another participant stressed the importance of flexibility, emphasizing the importance of being responsive to the learner as opposed to following a rigid procedure, stating, *“It’s not about the procedure; it’s about the child.”*

#### Flexible Trainers Make Compromises: Seeing the Bigger Learning Picture!

This subtheme represents a common thread among participants regarding the flexibility required to provide effective training in PT and promote high procedural fidelity in practice. Central to those perspectives was the view of intervention agents as learners. In seeing intervention agents as learners, participants highlighted the importance of compromise. Flexible trainers understand fidelity errors as learning opportunities that are part of the intervention agent’s implementation journey and accept that when PT is implemented in practice, it may not look like what the trainer initially envisioned or as described in textbooks or journal articles. Overall, the subtheme encompasses participants’ perspectives that trainers should view intervention agents, such as teachers, in the same way teachers view their learners.

*“Just remember that the principles of behavior analysis and the system of PT work for the people you’re training just as much as they work for the kids.”*

Participants believed that in their role as trainers, it is important to tolerate some fidelity errors to get intervention agents invested in the system. Participants suggested that in order to be a flexible trainer, one should acknowledge their biases.

“*You can be married to a science, but you also have to realize when people don’t like it.*”

*“Within our science, we’ve got to not come across as telling anybody this is a better way of doing things, and we know better than you.”*

Participants made reference to implementation being a social process, not solely a scientific process, even if the practice being implemented is scientific. The trainer merely telling an intervention agent to implement a procedure will not evoke implementation behavior. The participants brought attention to the social hierarchy between trainers and intervention agents and emphasized the need to avoid inflexible and perhaps authoritative approaches to implementation.

As trainers, participants recognized that errors may have benefits. Although this may sound counterintuitive, participants believed that allowing intervention agents to implement PT to a lower level of fidelity may be advantageous. Specifically, they suggested that if trainers are flexible and accept the flexibility of some components, intervention agents, such as teachers, may be more enthusiastic about using PT in their classrooms.

“*It won’t be as effective. It will still be very effective […] they find it easier. And actually, the practice and the timings do happen*.”

“*They (students) had their goals and so on, but they were not using the chart at all, and that was a lot better than not doing it*.”

Participants believed that tolerating some errors in implementation was “*better than nothing*” but recognized its shortcomings, stating “*it was missing like a lot at the same time*.”

Participants suggested that intervention agents may be more enthusiastic if trainers were flexible regarding certain fidelity errors, as this could reduce the perceived response effort associated with PT. Participants regularly referred to the high response effort involved in implementing PT as a teacher. As one participant pointed out, *“teachers are overworked,”* and the presentation of a high-response effort task such as engaging in training and implementing PT can lead teachers to ask, *“Why do I have to do more?”.* Participants emphasized the importance of trainers finding *“common ground”* with teachers and showing them how easy it is to fit into PT what they are already doing.

*“A lot of people will ask me ‘how do I start’ […] I go – pick one task, just pick one task that lends itself quite easily to doing this and see how you get on.”*

Participants believed that intervention agents viewed some components of the PT system as having higher response effort than others and that this may be where trainers need to be most flexible. They suggested a variety of strategies that could be used to address this. Participants highlighted that intervention agents believe the chart is high response effort and suggested that *“digitally charting”* could be an alternative to address this. Digitally charting involves using digital software to automatically plot the data on a virtual SCC once the user enters the number of correct and incorrect responses and the count time.

Data review and decision-making analysis were named by participants as other elements that teachers found demanding. One participant explained, *“For people that haven’t trained in behavior analysis or psychology, looking at data is something very, very overwhelming.”* Participants expressed that intervention agents often believe that they must *“carry a pile of charts home every night.”* However, participants identified that making decisions in the moment and having a system for decision-making was crucial to the sustainability of PT and was an effective way to reduce this perceived response effort.

*“This is not your evening activity. This is what you do right on the spot.”*

A few participants also suggested that having a learner become their own intervention agent or chart manager can reduce the response effort required by teachers. When the learner is the intervention agent, they can run their own timings, chart their own data, review data, and make decisions. This becomes a *“self-ownership procedure”* for learners, and teachers can take on a supervisory role by supporting students as they need. Participants also stressed that in order to reduce response effort for teachers, trainers should use accessible language, or “*plain English,”* as opposed to PT jargon as originally intended by Lindsley (1991).

*“It’s about using that language that’s familiar to them.”*

### It’s a System

The third theme comprises participants’ views of PT as a system and how it fits within other structures. Throughout the interviews, participants highlighted that the underlying principles are at the core, and the five components are built to form the PT system. Participants placed a lot of emphasis on the point that PT is more than an intervention or teaching methodology and that it is a series of components that must work in unison.

*“Everything goes so hand in hand, […] every step is important.”*

Participants stressed this point further by stating that although it is a flexible system, no component of the system could be eliminated entirely as each component builds on the previous. Participants recognized that often there are misconceptions that PT is an intervention for a specific problem or specific learner, that it is a flashcard procedure or frequency-building in isolation. Participants highlighted that these misconceptions lead to errors and more misconceptions, which can become a vicious cycle and are detrimental to the perception of PT.

*“The word gets out that Precision Teaching isn’t all that; it doesn’t work that well.”*

Participants differentiated PT from a specific intervention by emphasizing the bigger learning picture, that PT is a system to promote meaningful change in an individual’s life, teaching learners how to learn and grow.

*“There’s a whole method to teaching learners to grow. They’re learning to the point of automaticity […] It’s not just about charting things and turning timers on. It’s not just mechanics. […] there’s this big picture here.”*

Another element that participants mentioned that differentiates PT from an intervention is how it can be used with a variety of skills and used in conjunction with other interventions.

#### …Within a System

PT is one of many systems that are nested within a classroom system, school system, and the wider education system. Participants discussed how other systems and their structures can facilitate or act as a barrier to the implementation of PT with fidelity in practice. Participants described how classroom management issues may be a barrier to the implementation of PT in practice. However, strong classroom management systems and establishing strong session routines were identified by participants as a means to decrease the likelihood of other fidelity errors occurring.

*“So, there’s not good session routines in a classroom. Like the classroom is inefficient. It’s kind of unwieldy, like no… there isn’t this set way that the children are proceeding. So, it kind of gets looking kind of chaotic.”*

Participants highlighted the wider school environment as another system in which PT fits. Specifically, participants stressed the importance of a supportive school system to ensure its adoption in classrooms. If PT is not embedded within the school system, there may not be resources, including rooms and/or staff, to ensure effective implementation.

*“(If) it doesn’t get embedded, you know, teaching assistants are coming to us frustrated because they’re not getting the time to run those sessions, and you know they’ve turned up and the rooms occupied.”*

It was also believed that the hierarchy of intervention agents in the school system may not support implementing PT as intended. Participants stated that if teaching assistants implement PT sessions in schools, they may not have the authority to make changes to instruction approaches and may be required to bring this to the attention of the classroom teacher.

*“So, the systems sometimes don’t support some of the procedural aspects of a Precision Teaching model.”*

Participants also highlighted where PT fits within the wider education system. Participants reported clashes between the PT system and the education system with regard to curriculum pressures. Participants emphasized the importance of the time dedicated to practicing material within PT and achieving fluency in every skill before moving on. They also discussed how this contrasts with the education system model, which is focused on covering the curriculum.

*“We have a big pressure […] to reach some results, so we can test. […] They look at the end of the program, and so mostly to accuracy and not to frequency.”*

Participants also reported that the traditional training model offered through the education system does not facilitate the teaching of a large system such as PT, that is, *“There’s not a lot you can train in half a day without that kind of background knowledge.”* However, they suggested that if used in conjunction with in-class coaching, the traditional training model may be effective at training intervention agents to implement PT. This in-class coaching model fits within the participants’ discussion on the importance of an implementation support system. Participants stated that continued supervision or the chart parent concept within PT is vital to the sustainability of PT in practice. A chart parent refers to the person who has trained you to use the PT system and the SCC and becomes a mentor who supports you in implementing PT throughout the course of your PT experience.

*“Oftentimes people will make a ton of errors because they don’t have that… that frequent coaching and a chart parent to nurture their, their skillset and repertoire.”*

## Discussion

Procedural fidelity has long been an area of major discussion within ABA, with a range of literature exploring the impact of procedural fidelity on intervention outcomes (Carroll et al., 2013; Cook et al., 2015; DiGennaro Reed et al., 2011; Noell et al., 2002; Northup et al., 1997; St Peter Pipkin et al., 2010; Vollmer et al., 1999; Wilder et al., 2006). Yet, to our knowledge, this is the first study to explore the concept of procedural fidelity in PT. The authors adopted a novel approach influenced by the implementation science literature to develop an initial insight into the concept of procedural fidelity in the PT system, namely qualitative interviews and template thematic analysis.

Upon analysis of the interviews, three overarching themes related to procedural fidelity in PT were generated. The three themes include (a) The Complexity of Procedural Fidelity in PT, (b) Maintaining Fidelity without Losing Flexibility, and (c) It’s a System. The template thematic analysis was used to help the authors address the aims of the study: (a) explore the meaning of fidelity errors in the field of PT, (b) explore common fidelity errors witnessed when training and supervising teachers to use PT, and (c) explore the perceived impact of common fidelity errors on outcomes for learners. In addition to addressing the aims of the study, the authors used the template thematic analysis to analyze valuable information regarding how to shield against fidelity errors and promote high-fidelity implementation in PT.

### Explore Perspectives on the Meaning of Fidelity Errors in the Field of PT

Through thematic analysis, an insight into the meaning of a fidelity error in PT was developed; however, a clear definition warrants further exploration. The complexity of procedural fidelity and fidelity errors in PT is demonstrated by varying descriptions of fidelity errors across participants. The literature defines a fidelity error as adding something to a procedure that is not prescribed or omitting something that is prescribed (Breeman et al., 2020; DiGennaro Reed et al., 2011; St Peter Pipkin et al., 2010; Vollmer et al., 2008). While participants discussed fidelity errors that fit within this conceptualization, allowing the authors to classify errors as omission or commission errors throughout the dataset, the discourse highlighted an additional perspective that did not fit within this conceptualization. That is, participants described fidelity errors with regard to PT as strictly adhering to a procedure rather than following the underlying principles of the system and relying on a strong conceptual understanding to guide their implementation. Although this may seem counterintuitive, as fidelity refers to implementing a procedure as prescribed (St. Peter et al., 2023), participants believed it was more important to align with underlying principles of PT with less emphasis on making procedural errors as opposed to adhering strictly to a procedure while neglecting underlying principles such as the learner knows best. This is important to consider when training others to implement PT in that an emphasis should be placed on achieving fluency in underlying principles and essential core components while encouraging flexibility in elements of the procedure (e.g., decision-making, pinpointing) based on a sound knowledge of why and how the PT system works.

Participants’ view of the intervention agents as learners adds to the complexity of defining fidelity errors in PT. Participants mostly used the interviews as a place to advocate for flexibility and compromise. This aligns with recommendations from St. Peter et al. (2023) encouraging trainers to promote flexible and appropriate measurement of fidelity. In many cases, when providing training in PT, the trainer may work with teachers at a very early stage in their implementation journey, and as evidenced from the discourse in *“flexible trainers make comprises”* subtheme, they may be more lenient about the behaviors or approximations they accept in order to shape the intervention agents’ behavior.

As with many behavior analytic procedures, at the very core of the PT system is the individualization of approaches for each learner based on their ongoing performance, and this requires the use of intervention agents’ therapeutic and problem-solving repertoires, as well as their conceptual understanding, as opposed to the ability to follow pre-planned procedures. Research on behavior analytic supervision recommends against prescribing all elements, as this can result in intervention agents becoming insensitive to actual changes in the environment within sessions (Follette & Callaghan, 1995). On the basis of the current analysis, we loosely define fidelity to the PT system as adhering to the critical features or core components of the PT system with flexibility, ensuring that the underlying principle that the learner knows best is followed. We recommend that this definition be taken into consideration in conjunction with those put forth in the fidelity literature to date when implementing PT in practice. However, without experimental data, we cannot quantify the level of flexibility tolerable within the PT system.

### Explore Common Fidelity Errors Witnessed When Training and Supervising Teachers to Use PT

The views expressed throughout the interviews align with findings from descriptive studies suggesting that intervention agents do not consistently implement all components of a procedure with a high degree of fidelity and that some components may be subject to more fidelity errors than others (Breeman et al., 2020; Carroll et al., 2013; Foreman et al., 2021). These descriptive studies also highlight that a range of fidelity errors typically occur when procedures are implemented in practice, suggesting one reason why participants in the current study might have found it difficult to state errors that occur most often. Discussions about components that were subject to most fidelity errors centered on the *pinpoint*, *chart,* and *decide* components. The errors that were most commonly observed on the *chart* and *decide* components were errors of omission, specifically omitting the chart completely and not reviewing learning pictures. However, errors of commission were also discussed with regard to making decisions to change/add an intervention when learners were actually progressing. The pinpointing errors discussed were mostly errors of commission, with additional elements being added to pinpoints. One reason for fidelity errors occurring mostly within those components may be their distinct differences from typical behavioral interventions with which teachers or behavior analysts in schools might have previous experience. Intervention agents likely have a longer learning history with operational definitions and linear graphs, which are typically used in practice (Kubina et al., 2023, 2024).

Throughout the interviews, omission errors held a lot more space in the discourse compared with commission errors. However, when discussing the least common errors observed in practice, participants mentioned numerous commission errors. This echoes findings from the parametric analysis literature, which has shown more studies focused on errors of omission than commission (Brand et al., 2019), as well as observational studies, which highlight that more omission errors occur in practice (Foreman et al., 2021). The participants in the current study discussed the potential benefits of errors, which echoed the findings of Carroll et al. (2013) in which they found that the introduction of commission errors in the instruction component actually improved one learner’s performance. It is possible that commission errors were less dominant in the discourse as they are not perceived to be as detrimental to learners’ progress as omission errors. Differing opinions about which errors are most and least common among participants may be associated with differences in the PT training that participants offer, emphasizing the importance of research reporting on teacher training in PT.

### Explore the Perceived Impact of Common Fidelity Errors on Outcomes for Learners

Consistent with the literature, participants believed that, in general, higher levels of fidelity lead to better intervention outcomes (Brand et al., 2019). There was a strong belief across the dataset that errors on the *decide* component had the most detrimental impact on the effectiveness of the system, particularly when this component is omitted completely. Participants stressed that without reviewing learning pictures, one cannot assess the effectiveness of any given instruction or practice approach on learning outcomes. In other words, participants suggested omitting the *decide* component results in procedures no longer meeting the “analytic” dimension of ABA put forth by Baer et al. (1968). Participants spoke about the impact of errors not only in relation to academic progress but also on emotional well-being. They emphasized that without effective decision-making, learners and intervention agents may become frustrated and lose confidence. However, participants believed when implemented with fidelity, PT is associated with positive outcomes and can be, as one participant remarked, “*quite fun and exhilarating.”* This parallels previous PT literature that highlights beneficial outcomes associated with PT, such as increases in students’ self-esteem, motivation, concentration, self-fulfillment, and work habits (Beck & Clement, 1991; Boyce & Najdowski, 2003; Griffin & Murtagh, 2015; Sundhu & Kittles, 2016).

The findings from “Errors have a ripple effect” highlight the importance of looking at the whole PT system when considering error impact. Participants’ views suggest that an error in isolation may not appear detrimental; however, it may have knock-on effects on all the components to follow. This aligns with findings from Cook et al. (2015), that highlighted that global fidelity measures in isolation could mask the impact of component fidelity. If intervention agents implement all other components with a high degree of fidelity, the overall global fidelity score may be acceptable.

Although one of the underlying principles of PT is the display of data on the SCC (Lindsley, 1990), surprisingly, participants believed that errors in specific or more advanced charting conventions were not detrimental and that differentiation in symbols to display data was of less importance. Participants emphasized that provided frequency data is recorded and charted; trainers should be flexible regarding specifics. This aligns with participants’ perspectives that errors may have benefits, particularly in reducing perceived response effort. One participant believed that omitting the *chart* component completely in place of a typical line graph was not that damaging. A distinction can be drawn here between frequency-building alone and the PT system. While acknowledging that the benefits of frequency-building in the absence of the PT system are evidenced throughout the literature, it must also be acknowledged that without the chart, the procedure does not meet the critical components that are required to be classified as PT (Evans et al., 2021).

### Implications for Practice

The participants’ thoughts on the necessity for flexible intervention agents to maintain procedural fidelity are applicable to a number of interventions and approaches in ABA. Simply providing instructions to intervention agents describing how to implement a procedure can result in the intervention agent rigidly following the prescribed protocol regardless of what new problems may emerge in the session (Follette & Callaghan, 1995). Participants highlighted that training in PT should emphasize the underlying principles of the system and teach implementation skills to fluency. In turn, intervention agents will be able to adduce creative solutions to learning problems and be present and responsive to their learner’s needs. Such advice is generalizable to the wider field of behavior analysis, emphasizing the importance of conceptual training for intervention agents on the core principles and philosophical underpinnings of ABA.

The implementation challenges and fidelity errors emphasized throughout this study highlight areas for future trainers where intervention agents may need additional support and practice. With regard to implementing PT in practice, participants suggested that lower fidelity expectations, for example, charting weekly or using digital charts, may reduce response effort for teachers. This, in turn, should increase the sustainability of PT in practice. In recognition of the barriers and competing contingencies faced by intervention agents within the systems in which they operate, trainers should also consider a flexible approach to developing fidelity expectations and measures. Research has found that learning, although slower, can still occur at lower levels of fidelity (Brand et al., 2019), suggesting that there is a balance to be found between fidelity and acceptability. The answer to the question of where the balance is between fidelity and acceptability has yet to be answered and suggests a need for experimental analysis to examine further.

The “flexible trainers make compromises” subtheme also highlights the generality of the trainer’s behavior analytic skillset to their training role. Training should be developed on the basis of the principles of PT and ABA, and trainers should continue to follow these principles throughout the implementation process to shape intervention agent behavior to high levels of fidelity. The “It’s a system” theme reminds trainers to consider the constraints of the intervention agent’s environment while designing training. Proactively planning for competing contingencies such as resource availability and curriculum pressures may result in higher procedural fidelity and better intervention agent outcomes.

### Implications for Research

This study provides a preliminary insight into the concept of procedural fidelity in PT as perceived by experts in the field and highlights the importance of future research to develop a more in-depth understanding of this complex topic. Future researchers may develop this understanding through more qualitative research or other methods of inquiry. Specifically, researchers may benefit from exploring the perspectives of intervention agents who use PT in practice and comparing how their views align with expert teacher trainers. Future researchers may benefit from exploring the impact of fidelity errors across all phases of learning, including acquisition, fluency building, maintenance, and generalization. Descriptive assessments that gather real-time observational data on common procedural fidelity errors that occur in practice (Breeman et al., 2020; Carroll et al., 2013; Foreman et al., 2021) also offer an opportunity for future researchers to explore procedural fidelity in PT. Importantly, quantitative experiments such as parametric analyses are necessary to establish the true impact of fidelity errors on outcomes of PT. The findings from qualitative studies can inform the design of such studies in future research. More research in the area of procedural fidelity in PT will assist in developing standards regarding the minimum level of fidelity required on specific components of the PT system to maintain positive outcomes for learners.

The current findings also emphasize that PT does not occur in a vacuum and co-exists with a variety of other systems and with pre-existing values within the teacher’s classroom, the school, and the broader education system. As emphasized in the work of Slocum et al. (2014), the values of key stakeholders and context must be addressed to increase the uptake of evidence-based practices. Finding similarities between PT and school systems with regard to common values may facilitate the interweaving of PT into such systems and help it become embedded into the system’s culture. Addressing this in the literature and when implementing PT in practice may aid the successful implementation of PT and shield against potential fidelity errors that may occur owing to system clashes. In order for research to have a socially significant impact, future research could evaluate systems or interventions that have been co-designed with intervention agents and learners with their unique environment in mind.

### Strengths and Limitations

This paper provides valuable insight into expert’s views on procedural fidelity in PT. This paper is the first of our knowledge to explore procedural fidelity in PT and to take a novel approach to exploring fidelity using qualitative analysis. To our knowledge, the current study is the third qualitative study within the PT literature (Owen et al., 2021; Sundhu & Kittles, 2016). The findings highlighted the value and future potential of qualitative methods, including template thematic analysis in PT and ABA. However, this study is not without its limitations. First, the authors acknowledge that while qualitative studies are valuable for informing experimental analyses and, in this case, providing insights from experienced practitioners and researchers in PT, typically not accessible to intervention agents in practice, caution should be exercised when drawing conclusions from subjective data. Building from this research, controlled experimental studies should be conducted to establish, quantitatively, the impact of fidelity errors on PT outcomes. The authors recognize that the findings do not provide an exhaustive list of all the potential fidelity errors that may occur when using PT in practice. Another limitation of the study is that participants were not directly asked to discuss fidelity on each individual component of PT, and this may have resulted in participants failing to report some fidelity errors that they have witnessed in practice. The authors acknowledge the uneven distribution of participants across PT settings, with most working in both research and practice. Thus, views are not representative of individuals who use PT only in practice or research.

## Conclusion

Overall, this study provides an initial insight into procedural fidelity and fidelity errors in PT. Although the word precision appears in the title of the system, findings suggest “precision” does not mean without flexibility. Rather, Lindsley developed PT as a precise system of measurement that would facilitate teachers to evaluate instruction and curricula, fostering pragmatic analysis and leading to flexible, creative, and outcome-oriented teaching (Evans et al., 2021; West & Young, 1992). Participants’ views suggest that PT may be tolerant of some fidelity errors so long as the critical components and underlying principles are adhered to with fidelity. However, future research is warranted to explore the perimeters of when flexibility becomes a fidelity error.

## Data Availability

The datasets generated by the interviews and analyzed during the current study are stored open access on osf.io and are publicly available at the following link https://osf.io/n9q8zs/

## Appendix A – Interview Guide

### Section A: Experience with Precision Teaching

- Appropriately, how long have you used Precision Teaching in your research or practice?
- What is your experience training others to use Precision Teaching?
  o Who have you trained/what was their profession?
  o Tell me about the professional backgrounds the people you have trained come from.
  o Who were the learners that those you were training worked with?

### Section B: Implementation Errors in Practice

- What does a fidelity/implementation error in Precision Teaching mean to you?
- When training/supervising others using Precision Teaching, what implementation/fidelity errors do you commonly see?
  o Which of the fidelity errors mentioned do you believe are most common?
  o Which fidelity errors do you believe are least common?
- When you think of the Precision Teaching process and its parts, like *Pinpointing, Setting aims, Practice, Recording, Performance feedback and Decision Making Analysis* on which part/component do you see the most fidelity errors?
- What impact do you believe the fidelity errors you mentioned have on intervention efficacy?
  o Which implementation errors do you believe have the most detrimental/damaging impact on intervention efficacy?
  o Which fidelity errors do believe have the least impact on intervention efficacy?
  o On which component/ part of PT process *Pinpointing, Setting aims, Practice, Recording, Performance feedback and Decision Making Analysis* do you believe implementation errors are most & least detrimental?
- What are the most essential elements of the Precision Teaching process that you believe must be implemented to a high level of accuracy so that learners benefit from this approach?
  o Do you think that there are elements of the Precision Teaching process that could be eliminated or implemented poorly without greatly impacting outcomes for learners?
- Do you think there are elements of the PT approach that could be simplified/adapted to increase the adoption of PT into educational settings?
- What recommendations would you give to future trainers or supervisors who are training others to use Precision Teaching?
  o What’s the best approach to training teachers to use Precision Teaching?
  o What have you found worked/didn’t work?
- Is there anything else you would like to tell me about implementation accuracy or fidelity in Precision Teaching?
